# Supplementary material for: Large-Scale Data Mining of Rapid Residue Detection Assay Data From HTML and PDF Documents: Improving Data Access and Visualization for Veterinarians
Source: Front Vet Sci. 2021 Jul 21;8:674730. doi: 10.3389/fvets.2021.674730 (PMC8334182; doi:10.3389/fvets.2021.674730)
Supplement: Supplementary file 1 [file Table_1.DOCX]

Supplementary Material

# Supplementary Data

Data and codes associated with the analysis are available at <https://1data.life/pages/publication/Large%20scale%20data%20mining.html>.
